# Flap endonuclease 1 is involved in cccDNA formation in the hepatitis B virus

**DOI:** 10.1371/journal.ppat.1007124

**Published:** 2018-06-21

**Authors:** Kouichi Kitamura, Lusheng Que, Miyuki Shimadu, Miki Koura, Yuuki Ishihara, Kousho Wakae, Takashi Nakamura, Koichi Watashi, Takaji Wakita, Masamichi Muramatsu

**Affiliations:** 1 Department of Molecular Genetics, Kanazawa University Graduate School of Medical Sciences, Kanazawa, Japan; 2 Department of Radiology and Cancer Biology, Nagasaki University Graduate School of Biomedical Sciences, Nagasaki, Japan; 3 Department of Virology II, National Institute of Infectious Diseases, Tokyo, Japan; University of California, San Diego, UNITED STATES

## Abstract

Hepatitis B virus (HBV) is one of the major etiological pathogens for liver cirrhosis and hepatocellular carcinoma. Chronic HBV infection is a key factor in these severe liver diseases. During infection, HBV forms a nuclear viral episome in the form of covalently closed circular DNA (cccDNA). Current therapies are not able to efficiently eliminate cccDNA from infected hepatocytes. cccDNA is a master template for viral replication that is formed by the conversion of its precursor, relaxed circular DNA (rcDNA). However, the host factors critical for cccDNA formation remain to be determined. Here, we assessed whether one potential host factor, flap structure-specific endonuclease 1 (FEN1), is involved in cleavage of the flap-like structure in rcDNA. In a cell culture HBV model (Hep38.7-Tet), expression and activity of FEN1 were reduced by siRNA, shRNA, CRISPR/Cas9-mediated genome editing, and a FEN1 inhibitor. These reductions in FEN1 expression and activity did not affect nucleocapsid DNA (NC-DNA) production, but did reduce cccDNA levels in Hep38.7-Tet cells. Exogenous overexpression of wild-type FEN1 rescued the reduced cccDNA production in FEN1-depleted Hep38.7-Tet cells. Anti-FEN1 immunoprecipitation revealed the binding of FEN1 to HBV DNA. An *in vitro* FEN activity assay demonstrated cleavage of 5′-flap from a synthesized HBV DNA substrate. Furthermore, cccDNA was generated *in vitro* when purified rcDNA was incubated with recombinant FEN1, DNA polymerase, and DNA ligase. Importantly, FEN1 was required for the *in vitro* cccDNA formation assay. These results demonstrate that FEN1 is involved in HBV cccDNA formation in cell culture system, and that FEN1, DNA polymerase, and ligase activities are sufficient to convert rcDNA into cccDNA *in vitro*.

## Author summary

Hepatitis B virus (HBV) infection remains a worldwide health problem that affects more than 350 million people. HBV is one of the major etiological pathogens for liver cirrhosis and hepatocellular carcinoma. HBV covalently closed circular DNA (cccDNA) is a key viral intermediate for persistent infection. However, the molecular mechanism of cccDNA formation has not been clarified. Here, we found that the host factor flap-endonuclease 1 (FEN1) is pivotal in cccDNA formation. We developed a novel cccDNA formation assay by the incubation of purified viral DNA with recombinant FEN1, DNA polymerase, and DNA ligase. This study provides new insights into the molecular mechanisms of cccDNA formation and proposes FEN1 as a potential anti-HBV drug target.

## Introduction

Hepatitis B virus (HBV) is a major pathogenic cause of human cirrhosis and hepatocellular carcinoma [1]. Infectious HBV particles contain relaxed circular DNA (rcDNA) encapsidated by core proteins [2]. After entering the host hepatocyte, rcDNA is converted into covalently closed circular DNA (cccDNA), which is stably maintained as an episome in the nucleus. cccDNA serves as the template for all HBV transcripts, including pregenomic RNA (pgRNA), a viral replicative intermediate [2–4]. pgRNA, viral reverse transcriptase P protein, and core proteins assemble into a nucleocapsid, where pgRNA undergoes reverse transcription by the P protein to produce rcDNA. The mature nucleocapsid is further assembled with surface proteins to allow secretion as an infectious virion. Alternatively, the rcDNA containing nucleocapsid is recycled back to the nucleus to maintain the pool of cccDNA [5].

Reverse-transcriptase inhibitors are the major medical intervention for controlling HBV infection. These inhibitors can effectively shut down viral replication, but are unable to eliminate cccDNA from infected hepatocytes; this inability often leads to viral rebound upon therapy withdrawal [2, 3, 6]. New therapeutic approaches are needed to target the mechanisms of cccDNA maintenance and generation. However, a lack of comprehensive knowledge on the molecular mechanisms of cccDNA formation and maintenance has hampered the effective development of such approaches.

The cccDNA precursor rcDNA has unique structural features that are absent from cccDNA. These include a P protein-linked sequence approximately 10 nucleotides in length, known as terminal redundancy (r), which is located at the 5′ end of the minus-strand DNA, and a small RNA oligomer attached at the 5′ end of the plus strand [2, 6]. The first step in cccDNA conversion from rcDNA is removal of the P protein and RNA oligomer linkage from the 5′ ends. Resulting protein-free rcDNA or deproteinized rcDNA is proposed to be a direct precursor to cccDNA [7, 8]. In addition to removing the r sequence and RNA oligomer from rcDNA, filling-in the single-stranded region and ligation of nicks in both DNA strands are required for cccDNA formation.

Flap endonuclease 1 (FEN1) is a flap structure-specific endonuclease. FEN1 plays a role in removing 5′-flap structures formed during Okazaki fragment maturation and long-patch base excision repair (LP-BER) [9, 10]. Because the r sequence and RNA oligomer at the 5′ end of rcDNA may form a 5′-flap structure, we examined the possible involvement of FEN1 in the removal of 5′-flap structures from rcDNA and its subsequent conversion to cccDNA.

## Results

### The FEN1 inhibitor PTPD reduces HBV cccDNA levels

To determine whether FEN1 protein removes the r sequence from rcDNA, we designed a synthetic DNA substrate that mimics the 5′-flap structure of the r sequence (S1A Fig) by modifying an established FEN assay [11]. Human wild-type (wt) and catalytic mutant FEN1 proteins were prepared by immunoprecipitation (S1B Fig) and used for the HBV-FEN assay. Cleavage of the r sequence was determined by fluorescence intensity (S1C Fig) and polyacrylamide gel electrophoresis (PAGE) (S1D Fig). Incubation with immunoprecipitated FEN1 protein caused an increase in cleavage of the synthetic r sequence over time when compared with that of the mock-precipitated protein (S1C Fig). Conversely, two catalytic mutant FEN1 proteins [12] lost their cleavage activity (S1D Fig). Previous studies demonstrated the inhibition of flap endonuclease activity by the FEN1 inhibitor 3-hydroxy-5-methyl-1-phenylthieno[2,3-d]pyrimidine-2,4(1H,3H)-dione (PTPD) [11, 13]. In the current study, we examined whether PTPD could inhibit FEN activity of the immunoprecipitated FEN1 protein by using the HBV FEN assay. PTPD addition strongly inhibited FEN1 cleavage activity (S1E Fig).

**Fig 1:**
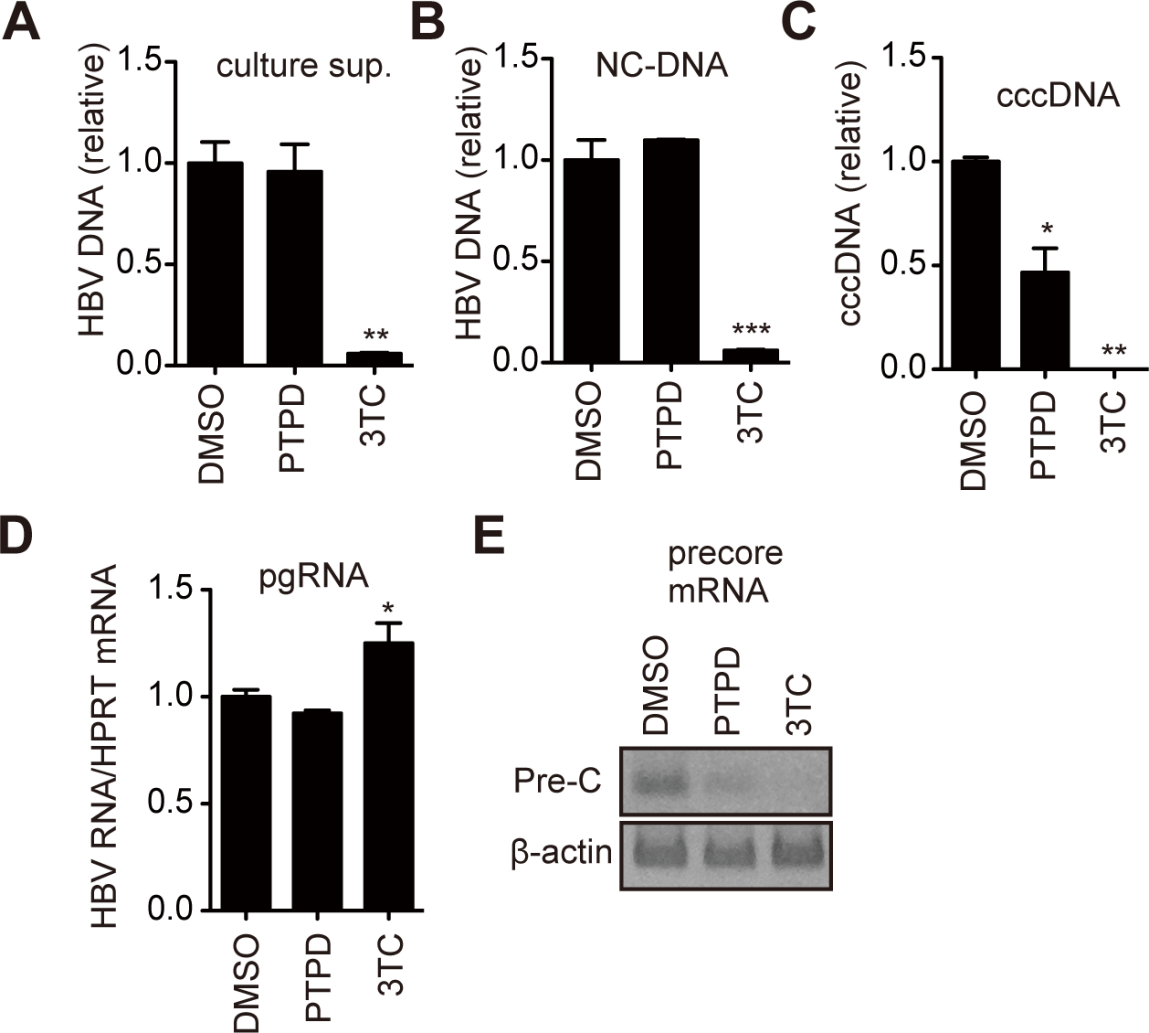
The FEN1 inhibitor, PTPD, reduces cccDNA production. Effect of FEN1 inhibition on HBV-replicating cells. Hep38.7-Tet cells were treated with dimethylsulfoxide (DMSO) as a vehicle control, PTPD (5 μM), or 3TC (50 μM) in the absence of tetracycline for 5 days. At day 5, levels of HBV DNA, HBV RNA (pgRNA normalized by HPRT) and pre-C mRNA were analyzed. qPCR analysis of HBV DNA in (A) culture supernatant, (B) cytoplasmic NC-DNA, (C) cccDNA, and (D) pgRNA. Each result represents the mean ± SEM of three independent experiments. Asterisks indicate statistically significant differences; *P < 0.05, **P < 0.01, ***P < 0.001 compared with DMSO (A–D). (E) RT-PCR analysis for pre-C mRNA transcribed from cccDNA in Hep38.7-Tet cells. β-actin was used as a loading control.

We used this inhibitor to explore the possible involvement of FEN1 in cccDNA formation in a cell culture system. Hep38.7-Tet cells replicate HBV and accumulate cccDNA after removing tetracycline from the culture medium [14, 15]. Using Hep38.7-Tet cells, the production of viral HBV intermediates was determined (Fig 1A–1E). Hirt extraction of cccDNA was followed by T5 exonuclease treatment to digest non-cccDNA molecules. T5 exonuclease removes nucleotides from 5′ termini, at gaps and nicks of linear or circular double-stranded DNA. The levels of cccDNA were determined by cccDNA-selective qPCR, which targets the gap region in rcDNA [16, 17] (Fig 1C). To demonstrate selective detection of cccDNA by the cccDNA-selective qPCR, a control experiment was performed. The Hirt extracted DNA and secreted HBV DNA were prepared from Hep38.7-Tet cells. The same copy number of the Hirt extracted DNA and secreted HBV DNA was applied to the cccDNA-selective qPCR. Our cccDNA-selective qPCR quantitatively detected cccDNA only from the Hirt-extracted HBV DNA, but not from secreted HBV DNA (S2 Fig).

To characterize the effect of PTPD treatment in Hep38.7-Tet cells, the effect of a reverse-transcriptase inhibitor, 3TC, was compared with that of PTPD. 3TC suppressed secreted and cytoplasmic nucleocapsid-associated DNA (cytoplasmic NC-DNA) and cccDNA levels (Fig 1A–1C). These results were expected, as HBV NC-DNA and cccDNA generation were completely dependent on reverse transcription in Hep38.7-Tet cells (S3 Fig), and 3TC was simultaneously added when Tet-CMV promoter was activated by removal of tetracycline from culture medium. Pre-C mRNA is transcribed from cccDNA, but not from the HBV transgene chromosomally integrated in cellular genome in the Hep38.7-Tet cells [18]. Consistent with the decreasing cccDNA levels in 3TC-treated Hep38.7-Tet cells, 3TC also reduced pre-C mRNA levels (Fig 1C and 1E). Conversely, PTPD significantly decreased both cccDNA and pre-C mRNA levels (Fig 1C and 1E). Importantly, PTPD did not affect the levels of secreted and cytoplasmic NC-DNAs (Fig 1A and 1B), confirming that transcription of pgRNA from the chromosomal copy was not affected by PTPD. Treating Hep38.7-Tet cells with PTPD (5 μM) for 5 days did not affect cellular proliferation (S4 Fig). These results suggest that PTPD blocked a step of cccDNA formation but did not inhibit reverse-transcription of pgRNA and cellular proliferation, as well as transcription from the HBV transgene.

### Reduction in FEN1 expression decreases cccDNA levels

**Fig 2:**
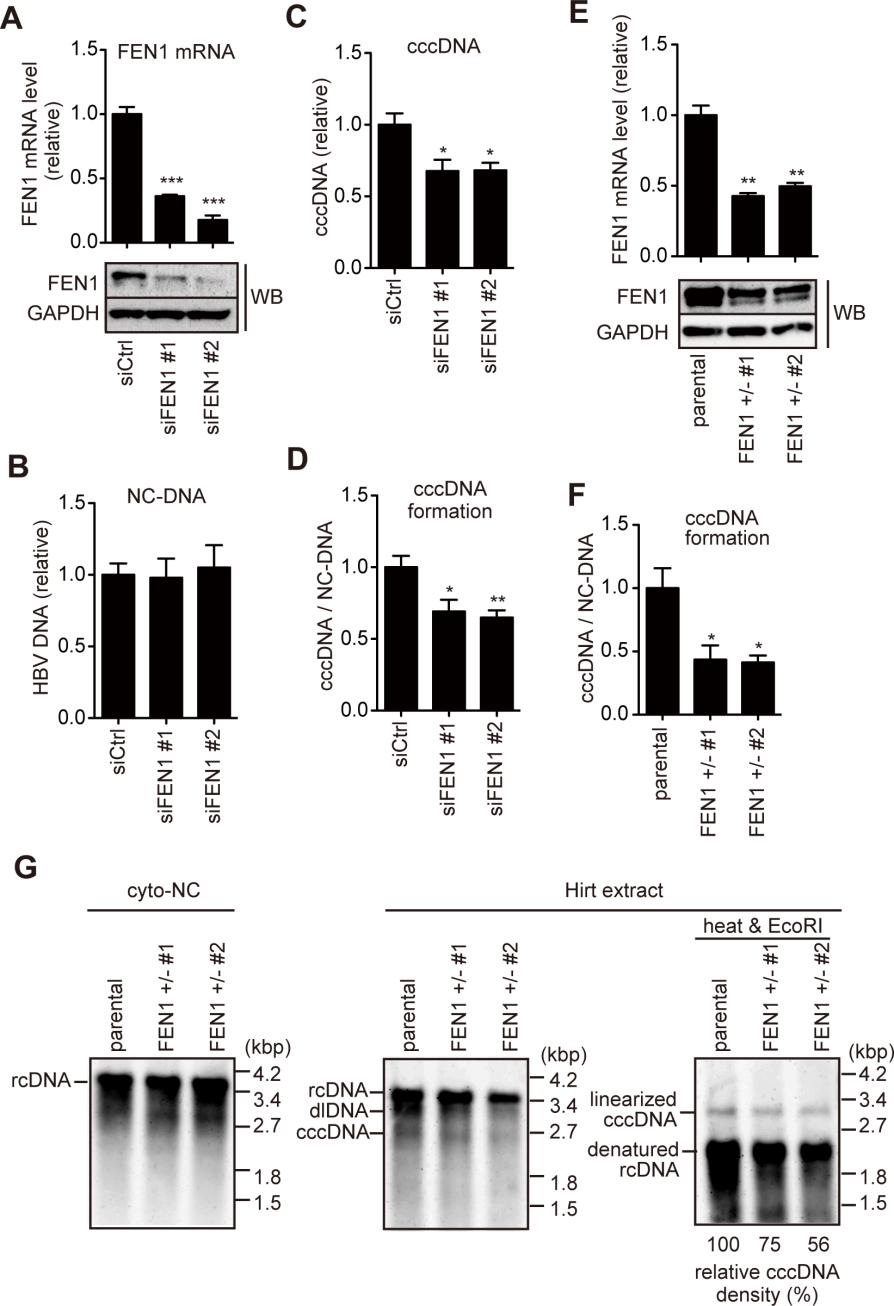
FEN1 siRNA knockdown and CRISPR/Cas9-mediated gene editing reduce cccDNA production. (A–D) Hep38.7-Tet cells were transfected with *FEN1*-specific siRNA (siFEN1 #1 or #2) or control (siCtrl), and cultured without tetracycline. Four days after transfection, FEN1 mRNA/protein and HBV DNA were analyzed. (A) FEN1 mRNA quantified by RT-qPCR (normalized by HPRT) (upper panel) and Western blotting of FEN1 protein (lower panel). GAPDH expression is shown as a loading control. FEN1 protein expression gives rise to two bands in our study, which may be due to post-translational modification. (B–C) Levels of HBV DNA in cytoplasmic nucleocapsid (NC) (B) and cccDNA (C). (D) Efficiency of cccDNA formation (cccDNA levels normalized by cytoplasmic NC-DNA) was calculated. Each result represents the mean ± SEM of three independent experiments. Asterisks indicate statistically significant differences; *P < 0.05, **P < 0.01, ***P < 0.001 compared with siCtrl. (E–G) The pX330-FEN1 plasmid, which expresses Cas9 mRNA and sgRNA for exon 2 of the *FEN1* gene, was transfected with a blasticidin-resistant gene expression vector into Hep38.7-Tet cells. After drug selection, two resistant clones (#1 and #2) were analyzed. (E) RT-qPCR and Western blotting analyses of FEN1 expression levels. (F) Efficiency of cccDNA formation. Each result represents the mean ± SEM of three independent experiments. Asterisks indicate statistically significant differences; *P < 0.05, **P < 0.01 compared with parental groups. (G) Southern blot analysis of cytoplasmic NC-DNA and Hirt-extracted HBV DNA. Hirt-extracted HBV DNA was analyzed with or without heat treatment and subsequent EcoRI digestion. Densitometric analysis of the EcoRI-digested cccDNA signal is shown below the Southern blot images. The signal for parental cell line is taken as 100%.

The FEN1 inhibitor experiments suggested that FEN1 is involved in cccDNA formation, but not rcDNA formation. However, the observed reduction of cccDNA level by PTPD was moderate, compared to that of 3TC (Fig 1C). To confirm this result by different approaches, we performed small interfering RNA (siRNA)-based knockdown and genome editing. Two siRNAs designed against FEN1 mRNA and control siRNA were transfected into Hep38.7-Tet cells. Transfection of FEN1 siRNA reduced FEN1 mRNA and protein expression by more than half of the control siRNA levels (Fig 2A). Consistent with the result in Fig 1, knockdown of FEN1 expression reduced, albeit moderately, the cccDNA levels without affecting the cytoplasmic NC-DNA level generated from the HBV transgene (Fig 2B and 2C).

To further confirm the requirement for FEN1 in cccDNA formation, CRISPR/Cas9-mediated genome editing was applied to Hep38.7-Tet cells. We obtained two independent lines of *FEN1*^*+/−*^ Hep38.7-Tet cells; each had one base (T) insertion in exon 2 of the *FEN1* gene. The one-base insertion caused a frame shift and premature stop codon at amino acid position 102, immediately after insertion (S5 Fig). RT-qPCR and Western blot analyses demonstrated reduced FEN1 expression up to approximately half of the parental Hep38.7-Tet cells (Fig 2E). Consistent with the results obtained from the knockdown experiments, *FEN1*^*+/−*^ Hep38.7-Tet cells produced cccDNA at approximately half the level of parental Hep38.7-Tet cells (Fig 2F). Southern blotting also showed moderately reduced cccDNA levels (Fig 2G, right), while intact cytoplasmic rcDNA production was observed in *FEN1*^*+/−*^ Hep38.7-Tet cells (Fig 2G, left). Taken together, the knockdown and genome editing results clearly demonstrated that reduced FEN1 expression decreased cccDNA levels without reducing cytoplasmic rcDNA levels in Hep38.7-Tet cells.

### FEN1 inhibitor blocks HBV replication in infection models

**Fig 3:**
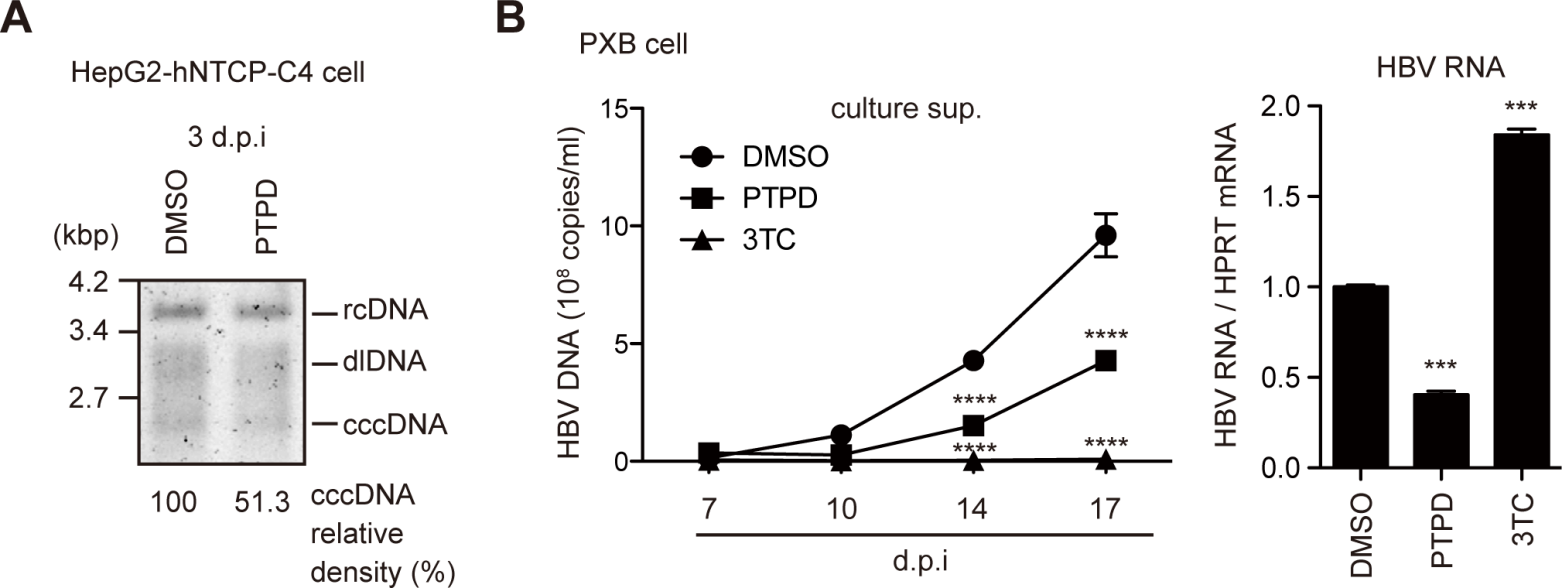
PTPD reduces HBV replication in HBV infection models. (A) HepG2-hNTCP-C4 cells were pretreated with PTPD (5 μM) for 1 day, and subsequently infected with HBV in PTPD-containing culture medium. cccDNA levels at 3 dpi were analyzed by Southern blotting. (B) PXB cells (primary human hepatocytes) were pretreated with PTPD (5 μM) or 3TC (50 μM) from 1 day before infection to 7 dpi. Secreted HBV DNA levels were quantified at the indicated time points (left side). HBV RNA was quantified by RT-qPCR at 21 dpi (right side). Asterisks indicate statistically significant differences; *P < 0.05 compared with DMSO.

Recent studies documented successful HBV infection in NTCP-expressing HepG2 cells [19, 20]. Thus, we examined the involvement of FEN1 in cccDNA formation using NTCP-expressing HepG2 (HepG2-hNTCP-C4) cells [19]. HepG2-hNTCP-C4 cells were pretreated with PTPD for 1 day, and subsequently infected with HBV. HBV-infected HepG2-hNTCP-C4 cells were cultivated for 3 days in the presence of PTPD, and cccDNA levels were determined by Southern blotting. As indicated in Fig 3A, the cccDNA level was mildly reduced by PTPD treatment (51.3% of control cccDNA level). Exposure of PTPD for 7 days in this cell line did not affect cellular proliferation (S6 Fig). To further confirm the results of infected HepG2-hNTCP-C4, we used PXB primary human hepatocytes derived from liver-humanized mice [21]. As shown in Fig 3B, PTPD treatment both inhibited secretion of HBV DNA and reduced HBV RNA levels in HBV-infected PXB cells. On the other hand, 3TC suppressed HBV DNA secretion but did not reduce HBV RNA levels. Importantly, in the infection model, rcDNA in the inoculum is first converted into cccDNA in the nucleus, and the newly produced cccDNA is transcribed into pgRNA, resulting in encapsidation and reverse-transcription in the nucleocapsid, yielding the mature virion (S3 Fig). Therefore, it is reasonable that PTPD treatment reduced both cccDNA formation and HBV DNA secretion in HBV-infected cells (Fig 3A and 3B). These results indicated that the FEN1 inhibitor blocks cccDNA formation following viral replication in infected NTCP-HepG2 cells and human primary hepatocytes.

### Mutant analysis of FEN1 in cccDNA formation

**Fig 4:**
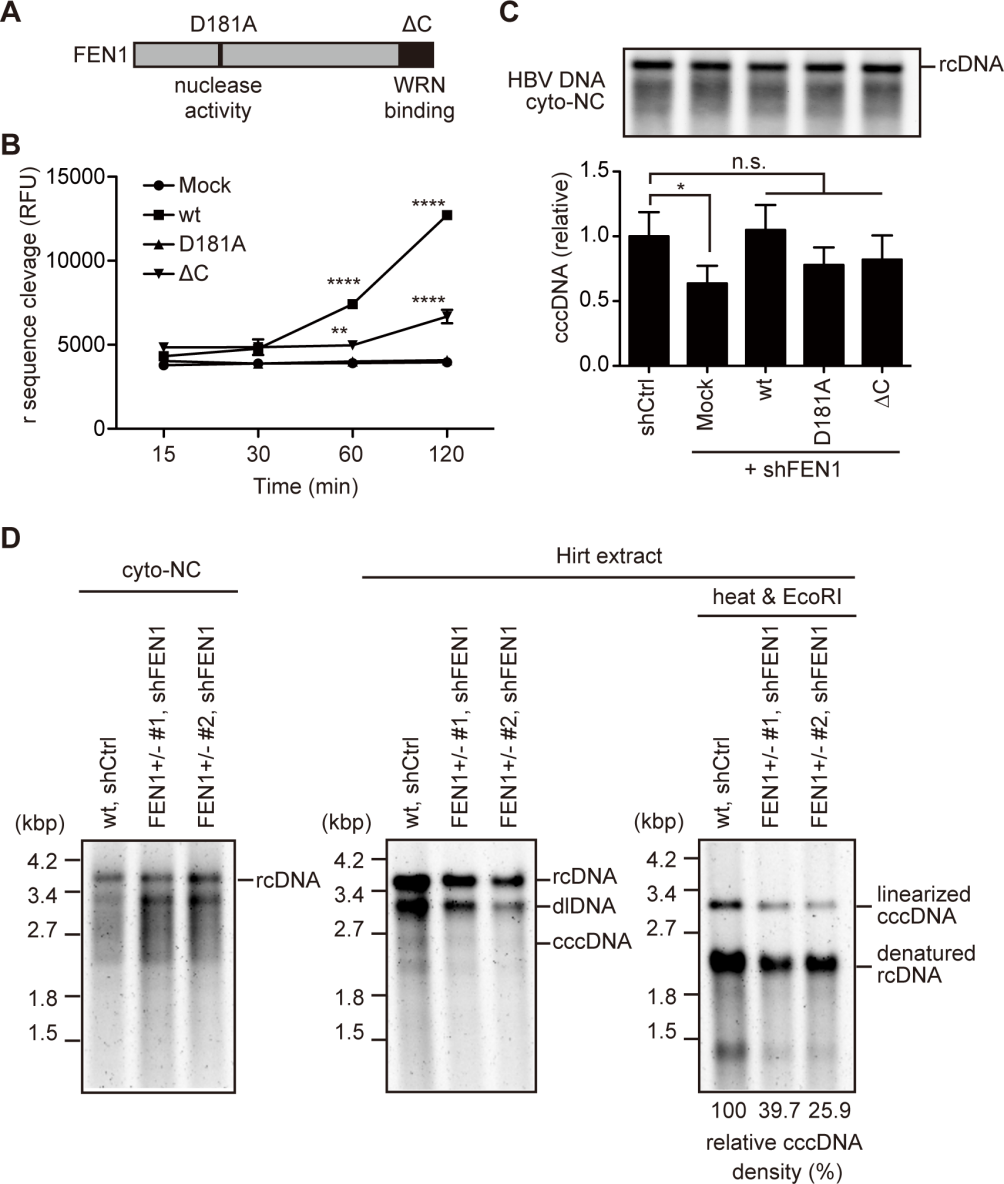
Requirement of nuclease activity and the C-terminus of FEN1 for cccDNA production. (A) Schematic presentation of FEN1 protein. D181A: nuclease-deficient mutant. ΔC: deletion mutant unable to bind WRN protein. (B) FEN assay. Flap endonuclease activity of immunoprecipitated FEN1 protein (wt, D181A, or ΔC) was determined as in Fig 1A. (C) pResQ lentiviral vectors carrying FEN1 shRNA and the FEN1 transgene (wt, D181A, or ΔC, see also S8 Fig) were transduced into Hep38.7-Tet cells. After puromycin selection, NC-DNA was analyzed by Southern blotting and qPCR, respectively. Each result represents the mean ± SEM of three independent experiments. Asterisks indicate statistically significant differences; ****P < 0.0001, **P < 0.01 compared with Mock (B), *P < 0.05 compared with shCtrl (C). (D) Two clones of the *FEN1*^*+/−*^ Hep38.7-Tet cells (#1 and #2 in Fig 2) were transduced with pResQ lentiviral vectors carrying FEN1 shRNA. wt, shCtrl. Hep38.7-Tet cells were transduced with the pResQ mock vector. After puromycin selection, FEN1 protein, cytoplasmic NC-DNA, and Hirt-extracted HBV DNA were analyzed by Southern blotting. Hirt-extracted HBV DNA was analyzed with or without heat treatment and subsequent EcoRI digestion. Densitometric analysis of the EcoRI-digested cccDNA signal is shown below the Southern blot images. The signal for the FEN1 wt cell line with control shRNA is considered 100%.

It was previously reported that a point mutation (D181A) of FEN1 results in a loss of nuclease activity, while deletion of 20 amino acids from the C-terminus (ΔC) of FEN1 results in a loss of binding to the telomere maintenance protein, WRN, and truncation of nuclear localization signal (Fig 4A) [22–24]. We first tested the FEN activity of these mutants with the HBV-FEN assay used in Fig 1. As demonstrated in Fig 4B, FEN1 wt protein cleaved the r sequence, while FEN1 ΔC showed lower cleavage activity and D181A could not cleave r. Importantly, all protein levels were comparable (S7 Fig).

To assess the requirement of nuclease activity and the C-terminus of FEN1 for cccDNA formation, we knocked-down endogenous FEN1 expression and simultaneously overexpressed either FLAG-tagged-wt, D181A, or ΔC FEN1 protein using the pResQ vector [25]. The pResQ lentiviral expression vector simultaneously expresses both short hairpin RNA (shRNA) targeting the 3′-untranslated region (UTR) of FEN1 mRNA (shFEN1) and exogenous wt or mutant FEN1 protein (S8A Fig). As shown in S8B Fig, endogenous FEN1 expression in shFEN1-transduced cells (mock, wt, D181A, ΔC) was significantly lower than in cells transduced with control shRNA (shCtrl). Furthermore, overall FEN1 expression levels in shFEN1-expressing wt, D181A, and ΔC transfectants were substantially higher than in shCtrl- and shFEN1-expressing mock transfectants, due to the exogenous expression of FEN1 protein. Western blotting confirmed the reduction of endogenous FEN1 and the expression of exogenous FEN1 protein, although endogenous FEN1 protein is visible in knockdown cells (S8C Fig). Cytoplasmic NC-DNA and cccDNA levels were also determined in these transfectants. All cells produced cytoplasmic NC-DNA at similar levels, while shFEN1-mock transfectants exhibited lower levels of cccDNA that were restored by wt FEN1 expression (Fig 4C). In addition, D181A and ΔC mutant transfectants tended to exhibit lower cccDNA levels than that of wt, although this tendency was not statistically significant (Fig 4C). Although these experiments do not conclusively demonstrate cccDNA formation roles for the FEN1 protein catalytic site and C terminus, they do show that FEN1 expression is required for cccDNA formation.

Since the reduction of the cccDNA level in *FEN1*^*+/−*^ Hep38.7-Tet cells was moderate (Fig 2G), we further examined the additive effect of genome editing and shRNA knockdown on cccDNA formation. pResQ shFEN1-mock lentiviral vector was transduced into two independent clones (#1 and #2) of *FEN1*^*+/−*^ Hep38.7-Tet cells. Endogenous FEN1 protein was effectively reduced in these *FEN1*^*+/−*^ shFEN1 cells (S8D Fig). Southern blotting analysis (Fig 4D) showed that *FEN1*^*+/−*^ shFEN1 cells produced cytoplasmic NC-DNA as effectively as shRNA control cells (shCtrl). Meanwhile, the cccDNA level was clearly reduced in *FEN1*^*+/−*^ shFEN1 cells compared with shRNA control cells (39.7 and 25.9% of the control cccDNA level, respectively). The additive effect of cccDNA reduction with the combination of *FEN1*^*+/−*^and FEN1 shRNA clearly indicates the requirement of FEN1 for cccDNA formation. It has been reported that rcDNA in Hirt extraction was reduced upon inhibition of cccDNA formation by knock-out of DNA ligases LIG1 and LIG3 [26]. It was proposed that nicked cccDNA behaves similar to rcDNA during electrophoresis, and a concurrent decrease of rcDNA may be due to a decrease in cccDNA formation. Reduction of rcDNA in Hirt DNA is also observed in our study (Figs 2G and 4D).

### FEN1 physically associates with HBV DNA in the nucleus

**Fig 5:**
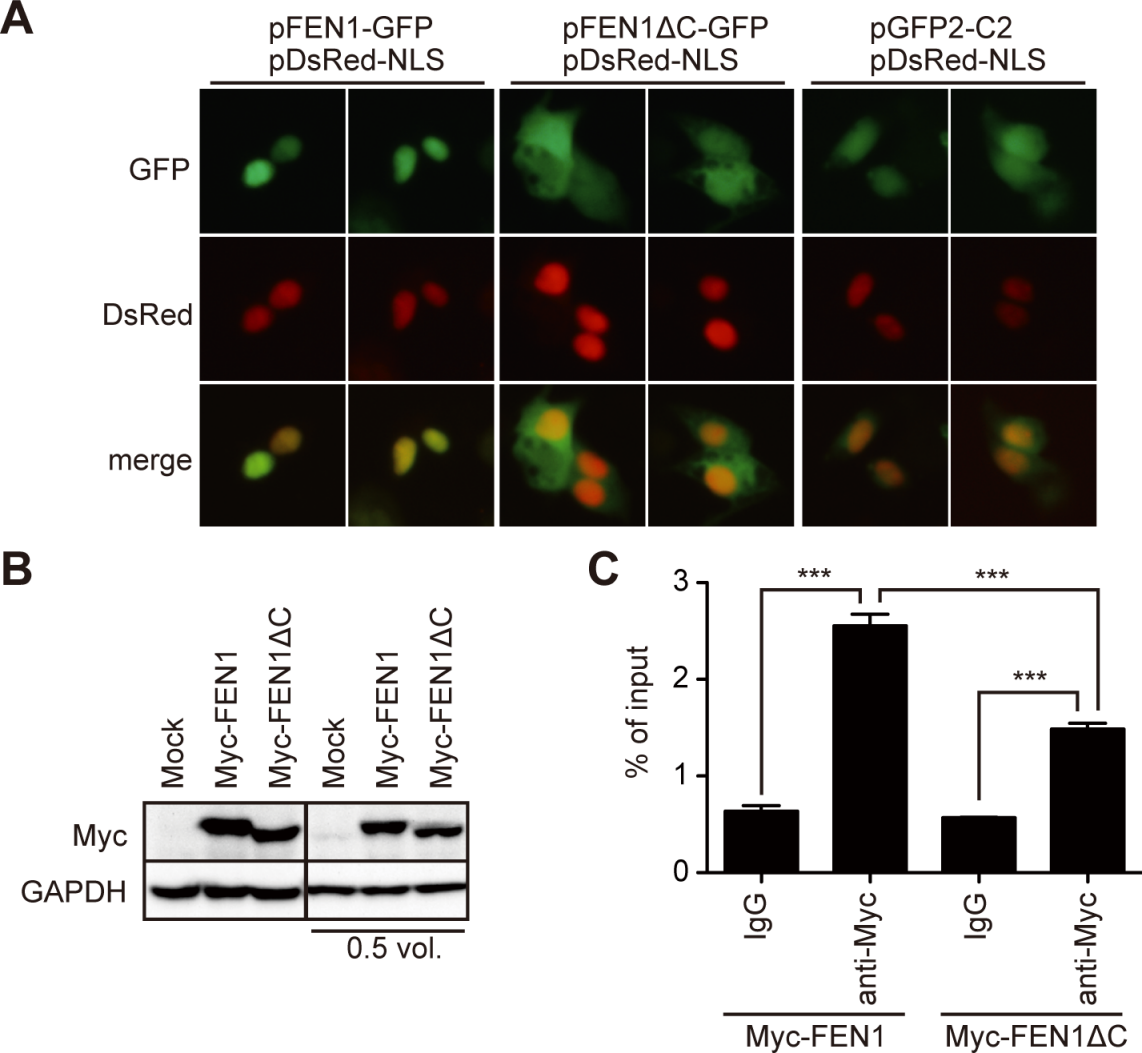
Deletion of the C-terminus disrupts the nuclear localization and reduces HBV DNA association of FEN1 protein. (A) Expression vectors of FEN1-GFP, FEN1ΔC-GFP, or mock vector (pcDNA4/TO) were transfected into Hep38.7-Tet cells. The nucleus was visualized by co-transfection of the nuclear localization signal (NLS)-tagged DsRed vector. (B–C) Myc-FEN1 or Myc-FEN1ΔC vector was transfected into Hep38.7-Tet cells. (B) Myc-tagged protein expression (before cross linkage) shown by Western blot. Two blots with different protein loadings are shown. (C) Myc-FEN1-transfected Hep38.7-Tet cells were cross-linked, and the lysates were immunoprecipitated with either control IgG or anti-Myc antibody. The immunoprecipitants were subjected to qPCR analysis using a primer pair to detect the core region of HBV. Each result represents the mean ± SEM of three independent experiments. Asterisks indicate statistically significant differences; ***P < 0.001.

Subcellular localization of FEN1 protein was examined in HBV-replicating Hep38.7-Tet cells. As expected [25], wt FEN1 protein localized to the nucleus, which was disrupted by the ΔC mutation (Fig 5A). We next utilized immunoprecipitation in order to determine whether FEN1 can associate with HBV DNA. c-Myc-tagged-wt or ΔC FEN1-expressing Hep38.7-Tet cells (Fig 5B) were treated with formaldehyde to cross-link protein and DNA, and FEN1 proteins were immunoprecipitated using a c-Myc antibody. The cross linkage and fragmentation of DNA that were necessary for this approach can make it difficult to judge which viral DNA forms are precipitated during FEN1 immunoprecipitation. However, the ability of FEN1 to associate with any of the viral DNAs can be estimated by comparison of the immunoprecipitated HBV DNA levels in the FEN1 with the control IgG conditions. As predicted, a significantly higher level of HBV DNA was detected in the FEN1 wt precipitate compared with that in the control as well as with the ΔC mutant precipitation (Fig 5C). Importantly, the ΔC mutant, missing its nuclear targeting ability, exhibited decreased HBV DNA binding, relative to the wild type FEN1 protein. This finding suggests that wild type FEN1 localizes to the nucleus and associates with nuclear HBV DNA, such as nuclear rcDNA, either directly or indirectly.

### *In vitro* cccDNA formation with recombinant FEN1

**Fig 6:**
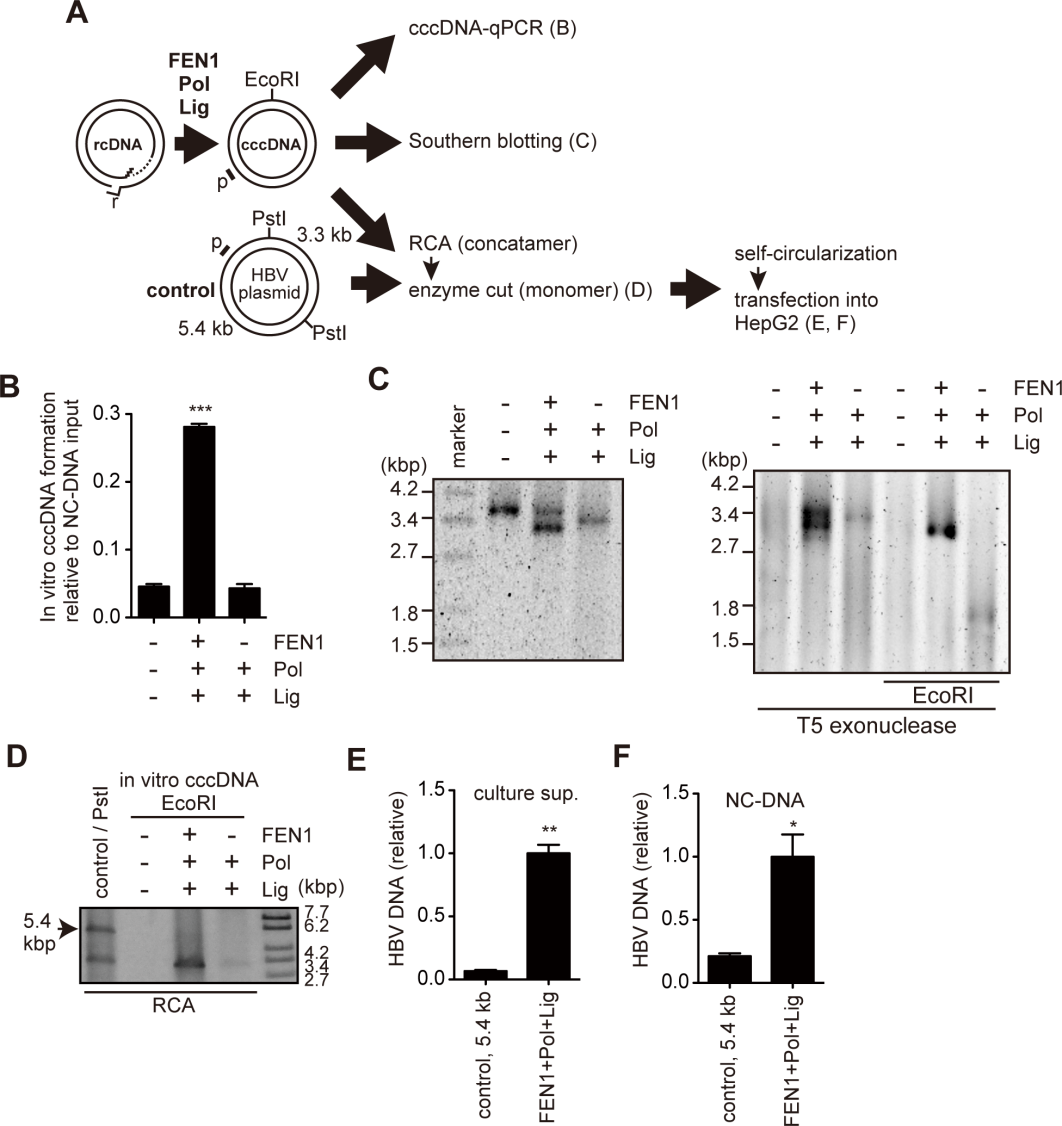
FEN1 protein facilitates cccDNA formation *in vitro*. (A) Schematic presentation of *in vitro* cccDNA formation assay. Purified NC-DNA (10^8^ copies) was incubated with recombinant FEN1, Bst DNA polymerase, and Taq DNA ligase. Following incubation, the DNA was purified and analyzed (B–F). Regions for qPCR amplification (E and F) were indicated as p. The 5.4-kb PstI fragment in HBV plasmid (Control) has a partial HBV sequence but does not have core and intact P genes. (B) cccDNA-selective qPCR. Each result represents the mean ± SEM of three independent experiments. Asterisks indicate statistically significant differences; ***P < 0.001 compared with negative control (no enzyme). (C) Southern blot analysis of *in vitro* cccDNA formation assay. The DNA was analyzed directly (left), or treated with T5 exonuclease (middle) or T5 exonuclease, then subsequently digested with EcoRI (right). (D) Detection of RCA products. DNA treated with indicated enzymes was subjected to RCA and then digested with indicated restriction enzymes. Arrow indicates the 5.4-kb fragment of the HBV plasmid, used as a replication-defective control for transfection in E and F. (E and F) Equal amounts of digested RCA product generated in (D) were self-circularized and then transfected into HepG2 cells. Three days after transfection, HBV DNA was analyzed by qPCR. Each result represents the mean ± SEM of three independent experiments. Asterisks indicate statistically significant differences compared with the control; * *P* < 0.05, ** *P* < 0.01.

Because FEN1 protein can remove the HBV r sequence *in vitro* (Fig 1A) and cellular experiments suggest a role of FEN1 in cccDNA formation (Figs 1–4), we assessed whether FEN1 can participate in any process of conversion of rcDNA to cccDNA *in vitro*. First, the FEN activity of recombinant FEN1 protein was reconfirmed by the HBV-FEN assay. Consistent with the results in Fig 1, recombinant FEN1 protein cleaved the r sequence from the synthetic HBV substrate in a dose-dependent manner (S9 Fig). Next, we determined whether recombinant enzymes, including FEN1, could convert the purified rcDNA into cccDNA. The purified NC-DNA from Hep38.7-Tet cells was incubated with recombinant FEN1, DNA polymerase, and DNA ligase, and cccDNA formation was determined by cccDNA selective-qPCR, rolling circle amplification (RCA), and Southern blot (Fig 6A–6D). RCA is able to speficically amplify closed circular DNA. The combination of FEN1, DNA polymerase, and DNA ligase led to the significant production of cccDNA (Fig 6B–6D). Meanwhile, incubation with two enzymes (DNA polymerase and DNA ligase) did not support efficient cccDNA formation (Fig 6B–6D). DNA sequencing of the closed circular DNA produced by three enzymes confirmed that the rcDNA gap region was precisely filled and did not have any mutations (S10 Fig). Furthermore, replication competency of in vitro-generated cccDNA was confirmed by transfecting the self-circularized RCA product into HepG2 cells (Fig 6E and 6F). These results indicate that the circular DNA generated by incubating with FEN1, DNA polymerase, and DNA ligase is functional HBV cccDNA.

## Discussion

Host DNA repair factors are expected to be involved in cccDNA conversion because the virus genome does not encode the responsible DNA modifiers [2, 3, 6, 27]. The TDP2 enzyme has been proposed to remove P protein [28], although another study reported that TDP2 is not required for cccDNA formation *in vivo* [29]. We previously showed that the host DNA repair enzyme UNG removes uracil residues from deaminated duck HBV (DHBV) cccDNA (or its precursor), thus changing its mutation frequency [30]. FEN1 plays a role in various DNA metabolic pathways, including Okazaki fragment maturation and LP-BER. During lagging strand DNA synthesis, Polδ/Polε use the strand exchange activity to produce the 5′-flap structure, and then FEN1 cleaves the 5′-flap and generates a ligatable end to facilitate lagging strand synthesis. In LP-BER, FEN1 removes the 5′-flap structure containing a damaged sugar and generates a ligatable 5′ end to facilitate its repair process [9, 10]. However, it remains unknown whether other back-up systems can substitute for absence of FEN1 activity.

During cccDNA formation, rcDNA-specific structures have to be removed. We assume that some of these rcDNA-specific structures form the 5′-flap structure. FEN1 is a good candidate to remove them. To test this, we utilized FEN1 loss-of-functional approaches in HBV-replicating cells, including a FEN1 inhibitor (Figs 1 and 3), siRNA and shRNA knockdown (Figs 2 and 4), and CRISPR/Cas9-mediated genome editing (Fig 2). The four different approaches of FEN1 loss of function showed the same trend, that is, a moderate reduction in cccDNA levels. We also utilized a combined approach of the genome editing and shRNA knockdown (Fig 4D). This method resulted in *FEN1*^*+/−*^-FEN1 shRNA Hep38.7-Tet cells with a clearly reduced cccDNA level. This reduction seemed to be specific to cccDNA because NC-DNA production was not reduced in Hep38.7-Tet cells, which could produce NC-DNA from genome integrated HBV transgene. We interpreted this reduced level of cccDNA as a specific phenotype of FEN1 loss-of-function, rather than off-target effects from each approach. Infection experiments showed that inhibition of FEN1 activity reduced HBV DNA secretion in NTCP-expressing cells and primary human hepatocytes (Fig 3). FEN1 protein could cleave the r sequence *in vitro* (Figs 4 and S1) and convert purified rcDNA into cccDNA along with DNA polymerase and ligase in vitro (Fig 6). Altogether, these results demonstrate, for the first time, that the host DNA repair factor FEN1 is involved in HBV cccDNA formation, at least in the experimental models used in this study.

The cccDNA was not eliminated completely, even when the combination approach for FEN1 loss-of-function was employed, suggesting the possibility of other redundant enzymes. FEN1 is a member of the RAD2/XPG structure-specific 5′-nuclease family [31, 32]. Among them, exonuclease 1 (Exo1) is another candidate to remove the HBV r sequence from rcDNA, because it has both 5′ to 3′ exonuclease activity and endonuclease activity of the 5′-flap structure. Moreover, yeast genetic studies suggested that Exo1 and FEN1 activities may have a redundant role [33, 34]. The human genome encodes another flap-structure specific endonuclease designated DNA2. DNA2 plays a role to resolve a flap structure during Okazaki fragment maturation in yeast [35]. Further studies are needed to determine the host players other than FEN1 that remove the flap structure from rcDNA.

Inhibition of FEN1 activity did not lead to an obvious reduction in proliferation, at least in the experimental conditions used in this study (S4 and S6 Figs). Consistent with our observation, it was reported that the PTPD inhibitor showed little or no effect on cell growth of the T24 bladder cell line, but increased sensitivity to a DNA damage agent, i.e. methyl methanesulfonate [13]. Moreover, FEN1 mutations that abrogate nuclease activity have been detected in lung cancers and corresponding knock-in mice are viable with autoimmune, chronic inflammatory, and cancer phenotypes [36]. Meanwhile another knock-in mice of FEN1 mutant (F343A and F344A) that lose ability to bind PCNA but retain nuclease activity, die at birth [37]. On the other hand, *FEN1*^−*/−*^mice, which have a complete knock-out of FEN1, have a lethality phenotype as early as embryonic day 3.5 [38]. Recent biochemical and genetic approaches identified more than 30 FEN1 associating proteins [32] including proteins involving in DNA replication, such as PCNA, apoptosis, telomere stability, post-transcriptional modification, and DNA repair. It is likely that complete loss of FEN1 protein in mammal manifests as a disturbance of cellular survival because both nuclease-dependent and -independent functions of FEN1 are disrupted. Meanwhile, inhibition of nuclease activity of FEN1 may not result in immediate disturbance of cellular proliferation. Consistent with this idea, the *FEN1*^−*/−*^ cell line was not established in this study, even by CRISPR/Cas9-mediated genome editing.

The HBV-FEN assay revealed that FEN1 could remove the r sequence from a synthetic HBV DNA flap substrate. Moreover, the combination of FEN1, DNA polymerase, and DNA ligase was sufficient to convert cccDNA from purified rcDNA *in vitro*. It has been reported that Polδ and Lig I cooperate with FEN1 in Okazaki fragment maturation, and Polβ and Lig III cooperate with FEN1 in LP-BER [10, 32]. However, the specific polymerase and ligase involved in HBV cccDNA formation remain unknown. Interestingly, the T5 exonuclease-resistant cccDNA (Fig 6C, top) migrated at approximately 3.4 kbp which is much higher position than that of cccDNA formed in infected hepatocytes, suggesting that its topology was different from cellular cccDNA. It is also possible for other additional factors such as topoisomerase and gyrase to be involved in cccDNA formation *in vivo*. Thus, further studies need to determine other host factors responsible for cccDNA formation.

In summary, we demonstrate that reduced FEN1 expression and activity decreases cccDNA levels, and that FEN1 protein can bind and cleave the 5′-flap structure of HBV rcDNA *in vitro* to facilitate cccDNA conversion. The data implicate FEN1 as a critical enzyme involved in HBV cccDNA formation.

## Materials and methods

### Cell culture

Hep38.7-Tet cells derived from the HepAD38 cell line (obtained from Dr. Christoph Seeger at Fox Chase Cancer Center, Philadelphia) [15], and HepG2-hNTCP-C4 cells derived from HepG2 cells (obtained from the JCRB Cell Bank) [19] were cultured as described previously. Hep38.7-Tet cells were cultured with 0.3 μg/ml tetracycline to terminate HBV transcription. HBV production was induced in the cells by incubation in a tetracycline-free medium. 293FT cells (purchased from Invitrogen) were cultured as described previously [39]. PXB primary human hepatocytes were derived from liver-humanized mice [21]. The culture medium was purchased from PhoenixBio. For FEN1 inhibition experiments, PTPD (3-hydroxy-5-methyl-1-phenylthieno[2,3-d]pyrimidine-2,4(1H,3H)-dione; Glixx Laboratories) [13] was added to the culture medium.

### HBV-FEN assay

The HBV-FEN assay was performed as described previously [11] with minor modifications. Wild-type (wt) and mutant human FEN1 proteins were produced by transfecting FEN1 expression vectors [12] (S1 Table) into 293FT cells and enriched by immunoprecipitation with anti-c-Myc agarose affinity gel (A7470; Sigma-Aldrich), as described below. The DNA substrate was prepared by annealing of “flap,” “quencher,” and “template” oligonucleotides containing the HBV sequences listed in S2 Table (also see S1A Fig). Since 5-carboxytetramethylrhodamine (TAMRA) is attached to the 5′ end of the r sequence corresponding to the flap oligonucleotide, cleavage of the flap oligonucleotide by FEN activity can be measured as increasing fluorescence. DNA substrates were incubated with either FEN1 immunoprecipitants at room temperature or recombinant protein Thermostable FEN1 (*Thermococcus* species 9°N origin; New England Biolabs) at 65°C. Kinetic fluorescence data were collected on PowerScan (DS Pharma Biomedical). Cleavage of the labeled, “flap” oligonucleotide was confirmed with 6 M urea/20% polyacrylamide gel electrophoresis (S1D Fig).

### qPCR and RT-qPCR

Purification of HBV DNA (supernatant, cytoplasmic NC-DNA, and cccDNA) and total RNA were described previously [30] with minor modification. HBV DNA in culture supernatant was extracted using a NucleoSpin kit (Takara) according to the manufacturer’s protocol. The purified HBV DNA from this fraction is designated as secreted HBV in this study. Viral DNAs from enveloped virions and naked capsids are included in this fraction. For cytoplasmic NC-DNA, the cells were lysed with buffer [10 mM Tris-HCl (pH 8.0), 1 mM EDTA, 1% NP-40, 8% sucrose, proteinase inhibitor cocktail (Roche)]. After centrifugation, supernatants were collected and further treated with DNase I and RNase A. NCs were then digested with proteinase K and sodium dodecyl sulfate (SDS). The cccDNA purification was performed using a modified Hirt extraction procedure [30]. The Hirt-extracted DNA was purified and treated with T5 exonuclease (New England Biolabs) to digest linear and open circular DNA according to the manufacturer’s instructions. Total RNA was treated with amplification grade DNase I (Thermo Fisher Scientific) and reverse transcribed using an oligo (dT) primer and the SuperScript III kit (Thermo Fisher Scientific). qPCR analysis of resulting cDNA was performed using SYBR green ROX (Toyobo) with MX3000 (Stratagene) as described previously [40]. For cccDNA quantification, TaqMan probe and cccDNA-selective primers spanning the gap region of rcDNA were used [16, 17]. Validation of selective amplification for cccDNA but not rcDNA, is shown in S2 Fig. Primers and probe sequences are listed in S2 Table.

### Rolling circle amplification (RCA)

RCA was performed as described previously [30, 41] using purified DNA from the Hirt extraction. In brief, the DNA (T5 exonuclease treated) was mixed with 8 HBV-specific primers (S2 Table), denatured at 95°C for 3 min; cooled sequentially at 50°C for 15 s, 37°C for 15 s, and room temperature, and reacted with the phi29 DNA polymerase (New England Biolabs) at 37°C for 16 h. RCA products were digested with EcoRI, which cuts HBV cccDNA once. The digested products were analyzed by gel electrophoresis and ethidium bromide staining.

### Immunoprecipitation and Western blotting

Immunoprecipitation and Western blotting were performed as described previously [30, 40]. To analyze FEN1-HBV DNA binding, cells were fixed with 1% formaldehyde for 10 min at room temperature, quenched with 125 mM glycine, resuspended in lysis buffer (50 mM Tris-HCl pH 8.0, 5 mM EDTA, 150 mM NaCl, 1% Nonidet P-40, 0.1% sodium dodecyl sulfate [SDS], protease inhibitor), sonicated in a Bioruptor sonication device (Diagenode) for 10 min using pulses of 30 s, and immunoprecipitated with anti-c-Myc antibody (9E10, sc-40; Santa Cruz Biotechnology) and protein G Sepharose (GE Healthcare) overnight at 4°C. Following proteinase K/SDS digestion, DNA was extracted with phenol/chloroform and precipitated with ethanol. Target DNA fragments were analyzed by qPCR as described above. The antibodies used for Western blotting were: mouse anti-FEN1 (4E7, GTX70185, GeneTex), rabbit anti-glyceraldehyde-3-phosphate dehydrogenase (GAPDH) (G9545; Sigma-Aldrich), mouse anti-FLAG (F3165, Sigma-Aldrich), mouse anti-c-Myc (9E10, sc-40; Santa Cruz Biotechnology), anti-rabbit Igs-horseradish peroxidase (HRP) (ALI3404; Biosource), and anti-rabbit/anti-mouse IgG-HRP TrueBlot (18–8816 and 18–8877; eBioscience).

### Southern blotting

Southern blotting was performed as described previously [30]. HBV DNAs were detected using a probe spanning the entire viral genome. Rhamda DNA probe was also simulateiously added to hybridzation buffer to visualize DNA size marker. Probe labeling and signal development was performed using the AlkPhos direct labeling system (Amersham), and the signals were detected using the LAS1000 imager system (Fuji Film). The Hirt-extracted DNAs were heat denatured at 95°C for 10 min and then subjected to EcoRI digestion to linearize DNAs. For the *in vitro* cccDNA formation (Fig 6C), HBV DNAs were treated with T5 exonuclease (New England Biolabs) to eliminate any DNAs, except double-stranded closed circular DNA. After phenol-chloroform extraction, DNAs were digested with EcoRI, and then agarose gel electrophoresis was performed.

### WST-1 assay

Cell viability was evaluated using the Premix WST-1 Cell Proliferation Assay System (Takara) according to the manufacturer's instructions. The cell lines used for the WST-1 assay were sensitive to puromycin; hence, puromycin was used as a control.

### siRNA transfection

Two *FEN1*-specific siRNAs and control siRNA were purchased from Santa Cruz Biotechnology (sc-37795, sc-37007) and Sigma-Aldrich (SASI_Hs02_00336939). Lipofectamine 3000 (Thermo Fisher Scientific) was used to perform transfections with these siRNAs according to the manufacturer’s instructions. Cells and viruses were analyzed 4 days after transfection.

### CRISPR/Cas9-mediated gene targeting

Human *FEN1*-targeting oligonucleotides (target sequence with the protospacer adjacent motif is in exon 2: 5′-AGCTGGCCAAACGCAGTGAGCGG-3′) were designed and cloned into the BbsI site of the pX330-U6-Chimeric_BB-CBh-hSpCas9 vector (a gift from Feng Zhang, Addgene plasmid # 42230) [42]. The resulting pX330-FEN1 vector was co-transfected into Hep38.7-Tet cells with pIRES-GFP-bsd, a blasticidin-resistant gene expression vector (S1 Table). Transfected clones were then selected using limiting dilution in the presence of blasticidin, and genome editing was confirmed by direct sequencing of the targeted region (oligonucleotides are listed in S2 Table).

### Lentivirus-mediated gene transduction

Lentivirus-mediated gene transduction was performed as described previously [40], using pResQ shFEN3 3XF-FEN1 wt, pResQ shFEN3 3XF-FEN1 D181A, and pResQ shFEN3 3XF-FEN1 ΔC (gifts from Sheila Stewart, Addgene plasmid # 17752, 17753, and 17754, respectively) [25]. Construction of pResQ vectors is described in S7A Fig and S1 Table.

### HBV infection

HBV infection was performed as described previously [19, 21]. Briefly, HBV (genotype D) was prepared from the culture supernatant of Hep38.7-Tet cells and concentrated with PEG8000 precipitation. The amount of HBV DNA was quantified by qPCR as described above. HepG2-hNTCP-C4 cells and PXB cells were seeded in collagen-coated plates, and the medium was replaced with fresh medium containing 4% PEG8000 and the prepared HBV (15,000 GE/cell for HepG2-hNTCP-C4 infection, 100 GE/cell for PXB infection). Twenty-four hours post-infection, the infected cells were washed three times with phosphate buffered saline and switched to fresh medium with PTPD or lamivudine (3TC). The cells and culture supernatants were collected at the indicated days post infection (d.p.i.).

### *In vitro* cccDNA formation assay

Purified NC-DNA from Hep38.7-Tet cells was used as substrate DNA. NC-DNA (10^8^ copies) was incubated with 32 units (U) of Thermostable FEN1 in ThermoPol Buffer (New England Biolabs) at 65°C for 10 min, followed by incubation with 8 U of Bst DNA polymerase, 40 U of Taq DNA ligase, 100 μM dNTPs, and NAD^+^ (all from New England Biolabs). After further incubation at 37°C for 20 min, DNA was purified by phenol/chloroform extraction and ethanol precipitation, and subjected to cccDNA-selective qPCR or RCA, as described above. The sequence corresponding to gap region of rcDNA was confirmed by direct sequencing (oligonucleotide is listed in S2 Table). To verify the replication competence of resulting products, EcoRI-digested RCA products (3.2 kb) were extracted from the gel; 50 ng of these fragments were subjected to self-circularization by T4 DNA ligase (Takara). For the negative control, HBV plasmid (pPB [30]) was amplified with RCA and then digested with PstI. Because the 5.4-kb PstI fragment has a partial HBV sequence, it was used as a replication-defective control. Self-circularized DNAs were transfected into HepG2 cells. Three days after transfection, HBV DNAs were analyzed by qPCR.

### Statistical analyses

Statistical analyses were performed using GraphPad Prism (GraphPad Software). Significance between two groups was determined using a Student’s *t*-test, while significance between three or more groups was determined using a one-way ANOVA with Dunnett's post-hoc test. P-values < 0.05 were considered statistically significant.

## Supporting information

S1 Fig HBV FEN assay. A fluorophore/quencher-labeled DNA substrate containing the 5′-flap structure of the HBV r sequence was incubated with immunoprecipitated Myc-FEN1 from 293FT transfectants (or immunoprecipitant from Mock-vector transfectant). Fluorescence signal (representing cleavage of the r sequence) was quantified. (A) Schematic presentation of DNA substrate. The substrate was prepared by annealing three synthesized oligonucleotides containing the HBV sequences. The complementary regions are indicated with lines. F represents the 5-TAMRA fluorophore, and Q represents the BHQ quencher. Cleavage of the 5´-flap structure (arrow) on the fluorophore-labeled oligonucleotide (red) results in release of fluorophore moiety from the quencher, and thus increases the fluorescence signal. (B) Western blotting for the immunoprecipitated Myc-tagged FEN1 proteins. FEN1 N has D181A mutation, whereas FEN1 NP has D181A, F343A, and F344A mutations. (C) Time course measurements of FEN1 activity. (D) After completion of the FEN fluorescence assay in C, cleavage of the flap structure was confirmed with urea-PAGE. Positions of substrate (31 nt) and cleaved product are indicated. (E) Inhibitory activity of PTPD was confirmed by the FEN assay. Asterisks indicate statistically significant differences compared with the control; **** *P* < 0.0001 compared with Mock (A).
(EPS)

S2 Fig Verification of the cccDNA-selective qPCR. HBV DNAs are purified from two fractions (culture supernatant, Hirt extraction) of Hep38.7-Tet cells. Hirt extracted DNA was further treated with T5 exonuclease. HBV DNA copy numbers from both fractions were determined by qPCR using primers indicated by p. To test selective amplification of cccDNA by our protocol, serial dilution of 1x10^5^ copy/μl from 1x up to x1/8 were prepared from both fractions, then the cccDNA-selective qPCR was performed.
(EPS)

S3 Fig HBV replication cycle in Hep38.7-Tet and infected HepG2-hNTCP-C4 cells. A proposed model for the pathway of cccDNA formation in HBV replication cycle. After entry through the NTCP receptor, the viral genome translocates into the nucleus, and rcDNA is converted to cccDNA. The cccDNA conversion comprises the following steps: removal of viral polymerase (P protein), removal of r sequence and RNA oligomer, completion of DNA synthesis in single-stranded region, and ligation of DNA ends. In Hep38.7-Tet cells, viral replication starts from the chromosomally integrated HBV transgene under the control of Tet-CMV promoter. 3TC, a reverse-transcriptase inhibitor, blocks the production of both rcDNA and cccDNA in Hep38.7-Tet cells. Meanwhile, infected HepG2-hNTCP-C4 cells can form cccDNA in the presence of 3TC. This study proposes that FEN1 is involved in the second step of cccDNA formation. Pre-C mRNA is transcribed from cccDNA but not directly from the HBV transgene.
(EPS)

S4 Fig WST-1 assay for PTPD-treated Hep38.7-Tet cells. Cell viability was evaluated by the WST-1 assay. Hep38.7-Tet cells were seeded into 96-well plates at 2,000 or 5,000 cells/well in the presence of PTPD (5 μM) for 5 days. Effect of puromycin (2 ng/ml) was compared with that of PTPD. Hep38.7-Tet cells are susceptible for puromycin. Each result represents the mean ± SEM of six independent experiments. Asterisks indicate statistically significant differences; ***P < 0.001 compared with negative control (DMSO).
(EPS)

S5 Fig Sequencing analyses of PCR products encompassing the *FEN1* target region. Results from two independent transfectants (#1 and #2) are shown. The mutation position is indicated by an asterisk. This T insertion causes a frame shift and generates a premature stop codon (underlined) in the *FEN1* open reading frame.
(EPS)

S6 Fig WST-1 assay for PTPD-treated HepG2-hNTCP-C4 cells. Cell viability was evaluated by the WST-1 assay. HepG2-hNTCP-C4 cells were seeded into 96-well plates at 500 or 1,000 cells/well, and treated with PTPD (2, 5, or 10 μM) for 7 days. Effect of puromycin (1 ng/ml) was compared with that of PTPD. HepG2-hNTCP-C4 cells are susceptible for puromycin. Each result represents the mean ± SEM of six independent experiments. Asterisks indicate statistically significant differences; ***P < 0.001 compared with negative control (0 μM).
(EPS)

S7 Fig Exogenous expression of FEN1 wt and mutant protein. Western blot analysis of myc-FEN1 (wt, D181A, ΔC) overexpression in 293FT cells and immunoprecipitants using anti-Myc beads. FEN activity of these immunoprecipitants was measured in Fig 5B.
(EPS)

S8 Fig FEN1 expression in pResQ-transduced Hep38.7-Tet cells. (A) Schematic presentation of pResQ vector construction. The lentiviral pResQ vector contains shRNA and transgene cassettes. shFEN1 targets the 3′UTR of endogenous FEN1 mRNA. Positions of FEN1 ORF primers used in B were indicated. (B) RT-qPCR to measure *FEN1* expression levels of pResQ-transduced Hep38.7-Tet cells. Levels of endogenous and total FEN1 mRNA were determined by qPCR (3´UTR and ORF, respectively). Each result represents the mean ± SEM of three independent experiments. Asterisks indicate statistically significant differences; **P < 0.01, ***P < 0.001 compared with shCtrl. (C) Western blot analysis of pResQ-transduced Hep38.7-Tet cells. The running time was longer than for Fig 2, in order to clearly separate the endogenous and exogenous FEN1 protein bands. (D) Western blot analysis of pResQ-transduced *FEN1*^*+/−*^ Hep38.7-Tet and control cells. GAPDH protein level is shown as a loading control.
(EPS)

S9 Fig FEN assay for recombinant FEN1. Verification of flap endonuclease activity in recombinant FEN1, using the HBV flap structure substrate from S1 Fig. Fluorescent signal (representing cleavage of the r sequence) was quantified. FEN1 Low: 32 U, FEN1 High: 144 U (per 50 μl reaction).
(EPS)

S10 Fig Sequencing analysis of cccDNA produced by *in vitro* cccDNA assay. Result from direct sequencing of the region corresponding to the gap of rcDNA is shown. (A) Chromatogram (B) Sequence alignment. The reference sequence (NCBI accession number U95551) is shown above. Asterisks in the alignment represent identity with the reference sequence. Positions of the r sequence, DR1, and DR2 are indicated.
(EPS)

S1 Table List of plasmids used in this study. (DOCX)

S2 Table List of oligonucleotides used in this study. (DOCX)

## References

[1] Trepo C, Chan HL, Lok A. Hepatitis B virus infection. Lancet. 2014;384(9959):2053–2063. doi: 10.1016/S0140-6736(14)60220-8 . 24954675 10.1016/S0140-6736(14)60220-8

[2] Seeger C, Mason WS. Molecular biology of hepatitis B virus infection. Virology. 2015;479–480:672–686. doi: 10.1016/j.virol.2015.02.031 ; PubMed Central PMCID: PMCPMC4424072. 25759099 10.1016/j.virol.2015.02.031 PMC4424072

[3] Guo JT, Guo H. Metabolism and function of hepatitis B virus cccDNA: Implications for the development of cccDNA-targeting antiviral therapeutics. Antiviral Res. 2015;122:91–100. doi: 10.1016/j.antiviral.2015.08.005 ; PubMed Central PMCID: PMCPMC4586118. 26272257 10.1016/j.antiviral.2015.08.005 PMC4586118

[4] Levrero M, Pollicino T, Petersen J, Belloni L, Raimondo G, Dandri M. Control of cccDNA function in hepatitis B virus infection. J Hepatol. 2009;51(3):581–592. doi: 10.1016/j.jhep.2009.05.022 . 19616338 10.1016/j.jhep.2009.05.022

[5] Kock J, Rosler C, Zhang JJ, Blum HE, Nassal M, Thoma C. Generation of covalently closed circular DNA of hepatitis B viruses via intracellular recycling is regulated in a virus specific manner. PLoS Pathog. 2010;6(9):e1001082 doi: 10.1371/journal.ppat.1001082 ; PubMed Central PMCID: PMCPMC2932716. 20824087 10.1371/journal.ppat.1001082 PMC2932716

[6] Nassal M. HBV cccDNA: viral persistence reservoir and key obstacle for a cure of chronic hepatitis B. Gut. 2015;64(12):1972–1984. doi: 10.1136/gutjnl-2015-309809 . 26048673 10.1136/gutjnl-2015-309809

[7] Gao W, Hu J. Formation of hepatitis B virus covalently closed circular DNA: removal of genome-linked protein. J Virol. 2007;81(12):6164–6174. doi: 10.1128/JVI.02721-06 ; PubMed Central PMCID: PMCPMC1900077. 17409153 10.1128/JVI.02721-06 PMC1900077

[8] Guo H, Jiang D, Zhou T, Cuconati A, Block TM, Guo JT. Characterization of the intracellular deproteinized relaxed circular DNA of hepatitis B virus: an intermediate of covalently closed circular DNA formation. J Virol. 2007;81(22):12472–12484. doi: 10.1128/JVI.01123-07 ; PubMed Central PMCID: PMCPMC2169032. 17804499 10.1128/JVI.01123-07 PMC2169032

[9] Liu Y, Wilson SH. DNA base excision repair: a mechanism of trinucleotide repeat expansion. Trends Biochem Sci. 2012;37(4):162–172. doi: 10.1016/j.tibs.2011.12.002 ; PubMed Central PMCID: PMCPMC3323758. 22285516 10.1016/j.tibs.2011.12.002 PMC3323758

[10] Henneke G, Friedrich-Heineken E, Hubscher U. Flap endonuclease 1: a novel tumour suppresser protein. Trends Biochem Sci. 2003;28(7):384–390. doi: 10.1016/S0968-0004(03)00138-5 . 12878006 10.1016/S0968-0004(03)00138-5

[11] Dorjsuren D, Kim D, Maloney DJ, Wilson DM, 3rd, Simeonov A. Complementary non-radioactive assays for investigation of human flap endonuclease 1 activity. Nucleic Acids Res. 2011;39(2):e11 doi: 10.1093/nar/gkq1082 ; PubMed Central PMCID: PMCPMC3025571. 21062821 10.1093/nar/gkq1082 PMC3025571

[12] Shibata Y, Nakamura T. Defective flap endonuclease 1 activity in mammalian cells is associated with impaired DNA repair and prolonged S phase delay. J Biol Chem. 2002;277(1):746–754. doi: 10.1074/jbc.M109461200 . 11687589 10.1074/jbc.M109461200

[13] Tumey LN, Bom D, Huck B, Gleason E, Wang J, Silver D, et al The identification and optimization of a N-hydroxy urea series of flap endonuclease 1 inhibitors. Bioorg Med Chem Lett. 2005;15(2):277–281. doi: 10.1016/j.bmcl.2004.10.086 . 15603939 10.1016/j.bmcl.2004.10.086

[14] Ladner SK, Otto MJ, Barker CS, Zaifert K, Wang GH, Guo JT, et al Inducible expression of human hepatitis B virus (HBV) in stably transfected hepatoblastoma cells: a novel system for screening potential inhibitors of HBV replication. Antimicrob Agents Chemother. 1997;41(8):1715–1720. ; PubMed Central PMCID: PMCPMC163991. 9257747 10.1128/aac.41.8.1715 PMC163991

[15] Ogura N, Watashi K, Noguchi T, Wakita T. Formation of covalently closed circular DNA in Hep38.7-Tet cells, a tetracycline inducible hepatitis B virus expression cell line. Biochem Biophys Res Commun. 2014;452(3):315–321. doi: 10.1016/j.bbrc.2014.08.029 . 25150444 10.1016/j.bbrc.2014.08.029

[16] Mason AL, Xu L, Guo L, Kuhns M, Perrillo RP. Molecular basis for persistent hepatitis B virus infection in the liver after clearance of serum hepatitis B surface antigen. Hepatology. 1998;27(6):1736–1742. doi: 10.1002/hep.510270638 . 9620351 10.1002/hep.510270638

[17] Watashi K, Liang G, Iwamoto M, Marusawa H, Uchida N, Daito T, et al Interleukin-1 and tumor necrosis factor-alpha trigger restriction of hepatitis B virus infection via a cytidine deaminase activation-induced cytidine deaminase (AID). J Biol Chem. 2013;288(44):31715–31727. doi: 10.1074/jbc.M113.501122 ; PubMed Central PMCID: PMCPMC3814766. 24025329 10.1074/jbc.M113.501122 PMC3814766

[18] Zhou T, Guo H, Guo JT, Cuconati A, Mehta A, Block TM. Hepatitis B virus e antigen production is dependent upon covalently closed circular (ccc) DNA in HepAD38 cell cultures and may serve as a cccDNA surrogate in antiviral screening assays. Antiviral Res. 2006;72(2):116–124. doi: 10.1016/j.antiviral.2006.05.006 . 16780964 10.1016/j.antiviral.2006.05.006

[19] Iwamoto M, Watashi K, Tsukuda S, Aly HH, Fukasawa M, Fujimoto A, et al Evaluation and identification of hepatitis B virus entry inhibitors using HepG2 cells overexpressing a membrane transporter NTCP. Biochem Biophys Res Commun. 2014;443(3):808–813. doi: 10.1016/j.bbrc.2013.12.052 . 24342612 10.1016/j.bbrc.2013.12.052

[20] Yan H, Zhong G, Xu G, He W, Jing Z, Gao Z, et al Sodium taurocholate cotransporting polypeptide is a functional receptor for human hepatitis B and D virus. Elife. 2012;1:e00049 doi: 10.7554/eLife.00049 ; PubMed Central PMCID: PMCPMC3485615. 23150796 10.7554/eLife.00049 PMC3485615

[21] Ishida Y, Yamasaki C, Yanagi A, Yoshizane Y, Fujikawa K, Watashi K, et al Novel robust in vitro hepatitis B virus infection model using fresh human hepatocytes isolated from humanized mice. Am J Pathol. 2015;185(5):1275–1285. doi: 10.1016/j.ajpath.2015.01.028 . 25791527 10.1016/j.ajpath.2015.01.028

[22] Sharma S, Sommers JA, Gary RK, Friedrich-Heineken E, Hubscher U, Brosh RM Jr. The interaction site of Flap Endonuclease-1 with WRN helicase suggests a coordination of WRN and PCNA. Nucleic Acids Res. 2005;33(21):6769–6781. doi: 10.1093/nar/gki1002 ; PubMed Central PMCID: PMCPMC1301591. 16326861 10.1093/nar/gki1002 PMC1301591

[23] Shen B, Nolan JP, Sklar LA, Park MS. Essential amino acids for substrate binding and catalysis of human flap endonuclease 1. J Biol Chem. 1996;271(16):9173–9176. . 8621570 10.1074/jbc.271.16.9173

[24] Stucki M, Jonsson ZO, Hubscher U. In eukaryotic flap endonuclease 1, the C terminus is essential for substrate binding. J Biol Chem. 2001;276(11):7843–7849. doi: 10.1074/jbc.M008829200 . 11083875 10.1074/jbc.M008829200

[25] Saharia A, Guittat L, Crocker S, Lim A, Steffen M, Kulkarni S, et al Flap endonuclease 1 contributes to telomere stability. Curr Biol. 2008;18(7):496–500. doi: 10.1016/j.cub.2008.02.071 ; PubMed Central PMCID: PMCPMC2367431. 18394896 10.1016/j.cub.2008.02.071 PMC2367431

[26] Long Q, Yan R, Hu J, Cai D, Mitra B, Kim ES, et al The role of host DNA ligases in hepadnavirus covalently closed circular DNA formation. PLoS Pathog. 2017;13(12):e1006784 doi: 10.1371/journal.ppat.1006784 ; PubMed Central PMCID: PMCPMC5747486. 29287110 10.1371/journal.ppat.1006784 PMC5747486

[27] Sohn JA, Litwin S, Seeger C. Mechanism for CCC DNA synthesis in hepadnaviruses. PLoS One. 2009;4(11):e8093 doi: 10.1371/journal.pone.0008093 ; PubMed Central PMCID: PMCPMC2778999. 19956651 10.1371/journal.pone.0008093 PMC2778999

[28] Koniger C, Wingert I, Marsmann M, Rosler C, Beck J, Nassal M. Involvement of the host DNA-repair enzyme TDP2 in formation of the covalently closed circular DNA persistence reservoir of hepatitis B viruses. Proc Natl Acad Sci U S A. 2014;111(40):E4244–4253. doi: 10.1073/pnas.1409986111 ; PubMed Central PMCID: PMCPMC4209993. 25201958 10.1073/pnas.1409986111 PMC4209993

[29] Cui X, McAllister R, Boregowda R, Sohn JA, Cortes Ledesma F, Caldecott KW, et al Does Tyrosyl DNA Phosphodiesterase-2 Play a Role in Hepatitis B Virus Genome Repair? PLoS One. 2015;10(6):e0128401 doi: 10.1371/journal.pone.0128401 ; PubMed Central PMCID: PMCPMC4469307. 26079492 10.1371/journal.pone.0128401 PMC4469307

[30] Kitamura K, Wang Z, Chowdhury S, Simadu M, Koura M, Muramatsu M. Uracil DNA glycosylase counteracts APOBEC3G-induced hypermutation of hepatitis B viral genomes: excision repair of covalently closed circular DNA. PLoS Pathog. 2013;9(5):e1003361 doi: 10.1371/journal.ppat.1003361 ; PubMed Central PMCID: PMCPMC3656096. 23696735 10.1371/journal.ppat.1003361 PMC3656096

[31] Harrington JJ, Lieber MR. Functional domains within FEN-1 and RAD2 define a family of structure-specific endonucleases: implications for nucleotide excision repair. Genes Dev. 1994;8(11):1344–1355. . 7926735 10.1101/gad.8.11.1344

[32] Zheng L, Jia J, Finger LD, Guo Z, Zer C, Shen B. Functional regulation of FEN1 nuclease and its link to cancer. Nucleic Acids Res. 2011;39(3):781–794. doi: 10.1093/nar/gkq884 ; PubMed Central PMCID: PMCPMC3035468. 20929870 10.1093/nar/gkq884 PMC3035468

[33] Sun X, Thrower D, Qiu J, Wu P, Zheng L, Zhou M, et al Complementary functions of the Saccharomyces cerevisiae Rad2 family nucleases in Okazaki fragment maturation, mutation avoidance, and chromosome stability. DNA Repair (Amst). 2003;2(8):925–940. . 12893088 10.1016/s1568-7864(03)00093-4

[34] Tishkoff DX, Boerger AL, Bertrand P, Filosi N, Gaida GM, Kane MF, et al Identification and characterization of Saccharomyces cerevisiae EXO1, a gene encoding an exonuclease that interacts with MSH2. Proc Natl Acad Sci U S A. 1997;94(14):7487–7492. ; PubMed Central PMCID: PMCPMC23848. 9207118 10.1073/pnas.94.14.7487 PMC23848

[35] Wanrooij PH, Burgers PM. Yet another job for Dna2: Checkpoint activation. DNA Repair (Amst). 2015;32:17–23. doi: 10.1016/j.dnarep.2015.04.009 ; PubMed Central PMCID: PMCPMC4522331. 25956863 10.1016/j.dnarep.2015.04.009 PMC4522331

[36] Zheng L, Dai H, Zhou M, Li M, Singh P, Qiu J, et al Fen1 mutations result in autoimmunity, chronic inflammation and cancers. Nat Med. 2007;13(7):812–819. doi: 10.1038/nm1599 . 17589521 10.1038/nm1599

[37] Zheng L, Dai H, Qiu J, Huang Q, Shen B. Disruption of the FEN-1/PCNA interaction results in DNA replication defects, pulmonary hypoplasia, pancytopenia, and newborn lethality in mice. Mol Cell Biol. 2007;27(8):3176–3186. doi: 10.1128/MCB.01652-06 ; PubMed Central PMCID: PMCPMC1899923. 17283043 10.1128/MCB.01652-06 PMC1899923

[38] Kucherlapati M, Yang K, Kuraguchi M, Zhao J, Lia M, Heyer J, et al Haploinsufficiency of Flap endonuclease (Fen1) leads to rapid tumor progression. Proc Natl Acad Sci U S A. 2002;99(15):9924–9929. doi: 10.1073/pnas.152321699 ; PubMed Central PMCID: PMCPMC126601. 12119409 10.1073/pnas.152321699 PMC126601

[39] Ahasan MM, Wakae K, Wang Z, Kitamura K, Liu G, Koura M, et al APOBEC3A and 3C decrease human papillomavirus 16 pseudovirion infectivity. Biochem Biophys Res Commun. 2015;457(3):295–299. doi: 10.1016/j.bbrc.2014.12.103 . 25576866 10.1016/j.bbrc.2014.12.103

[40] Liang G, Liu G, Kitamura K, Wang Z, Chowdhury S, Monjurul AM, et al TGF-beta suppression of HBV RNA through AID-dependent recruitment of an RNA exosome complex. PLoS Pathog. 2015;11(4):e1004780 doi: 10.1371/journal.ppat.1004780 ; PubMed Central PMCID: PMCPMC4383551. 25836330 10.1371/journal.ppat.1004780 PMC4383551

[41] Margeridon S, Carrouee-Durantel S, Chemin I, Barraud L, Zoulim F, Trepo C, et al Rolling circle amplification, a powerful tool for genetic and functional studies of complete hepatitis B virus genomes from low-level infections and for directly probing covalently closed circular DNA. Antimicrob Agents Chemother. 2008;52(9):3068–3073. doi: 10.1128/AAC.01318-07 ; PubMed Central PMCID: PMCPMC2533481. 18606836 10.1128/AAC.01318-07 PMC2533481

[42] Cong L, Ran FA, Cox D, Lin S, Barretto R, Habib N, et al Multiplex genome engineering using CRISPR/Cas systems. Science. 2013;339(6121):819–823. doi: 10.1126/science.1231143 ; PubMed Central PMCID: PMCPMC3795411. 23287718 10.1126/science.1231143 PMC3795411

