# Supplementary material for: Flap endonuclease 1 is involved in cccDNA formation in the hepatitis B virus
Source: PLoS Pathog. 2018 Jun 21;14(6):e1007124. doi: 10.1371/journal.ppat.1007124 (PMC6013022; doi:10.1371/journal.ppat.1007124)
Supplement: S1 Table — (DOCX) [file ppat.1007124.s011.docx]

**S1 Table. Plasmid list**

| **Plasmid** | **Description** | **Reference** |
| --- | --- | --- |
| pcDNA4/TO hFEN-wt | Expression vector of myc-His tag FEN1 wt | [1] |
| pcDNA4/TO hFEN-N | Expression vector of myc-His tag FEN1 nuclease-dead D181A mutant | [1] |
| pcDNA4/TO hFEN-NP | Expression vector of myc-His tag FEN1 mutant containing D181A, F343A and F344A. | [1] |
| pcDNA4/TO hFEN-ΔC | Synthetic olgonucleotides (1643,1644 see supplementary table 2) were hybridized to make a doube strand DNA linker. The 1643/1644 DNA linker was replaced with the C terminal DNA in FEN1 ORF using an EcoO65I/XbaI site in the pcDNA4/TO hFEN-wt, which results in generation of an expression vector for FEN1 deteting 21 amino acids from the C terminus of the FEN1 ORF. | this study |
| pcDNA4/TO mock | Obtained from Invitrogen |  |
| pResQ shCtrl | The FEN1 ORF and shFEN1 were deleted from pResQ shFEN1 wt. | this study |
| pResQ shFEN1 | The FEN1 ORF was deleted from pResQ shFEN1 wt. | this study |
| pResQ shFEN1 wt | A lentivirus vector for expression of both shFIN1 and Flag-tagged wt FEN1. pResQ shFEN3 3XF-FEN1 wt was a gift from Sheila Stewart (Addgene plasmid # 17752). | Addgene 17752 |
| pResQ shFEN1 D181A | A lentivirus vector for expression of both shFEN1 and Flag-tagged D181A FEN1. pResQ shFEN3 3XF-FEN1 D181A was a gift from Sheila Stewart (Addgene plasmid # 17753) | Addgene 17753 |
| pResQ shFEN1 ΔC | A lentivirus vector for expression of both shFIN1 and Flag-tagged FEN1 (C terminus 20 amino acids deletion). pResQ shFEN3 3XF-FEN1 DeltaC was a gift from Sheila Stewart (Addgene plasmid # 17754) | Addgene 17754 |
| psPAX2 | A helper plasmid to make a recombinant lentivirus. psPAX2 was a gift from Didier Trono (Addgene plasmid # 12260) | Addgene12260 |
| pMD2G | A helper plasmid to make a recombinant lentivirus. pMD2.G was a gift from Didier Trono (Addgene plasmid # 12259) | Addgene12259 |
| pX330 | pX330-U6-Chimeric_BB-CBh-hSpCas9 was a gift from Feng Zhang (Addgene plasmid # 42230) | Addgene 42230 |
| px330-FEN1 gRNA | Synthetic olgonucleotides (CACCGAGCTGGCCAAACGCAGTGAG, AAACCTCACTGCGTTTGGCCAGCTC) were hybridized to make a doube strand DNA linker. The DNA linker was inserted into BbsI site of pX330. | this study |
| pIRES-GFP-bsd | BstXI/NotI of blasticidin-resistant gene cassette was replaced with an EGFP gene in pIRES2-EGFP (CLONTECH) vector and designated pIRES-bsr, and then ExoRI/XhoI 0.7kb GFP2 fragment from pGFP2N3 (BioSignal Packard) was subcloned into an EcoRI/SalI site of pIRES-bsr. | this study |
| phFEN-GFP | The HindIII/SacII FEN fragment in pcDNA4/TO/hFENwt was subcloned into pGFP2-N3. | this study |
| phFENΔC-GFP | The HindIII/SacII FEN fragment in pcDNA4/TO/hFENΔC was subcloned into pGFP2-N3. | this study |
| pDsRed-NLS | An expression vector for NLS-tagged DsRed. | [2] |

Supplementary references

1. Shibata Y & Nakamura T (2002) Defective flap endonuclease 1 activity in mammalian cells is associated with impaired DNA repair and prolonged S phase delay. J Biol Chem 277(1):746-754.
2. Chowdhury S, Kitamura K, Simadu M, Koura M, & Muramatsu M (2013) Concerted action of activation-induced cytidine deaminase and uracil-DNA glycosylase reduces covalently closed circular DNA of duck hepatitis B virus. *FEBS Lett* 587(18):3148-3152.
