# Supplementary material for: Flap endonuclease 1 is involved in cccDNA formation in the hepatitis B virus
Source: PLoS Pathog. 2018 Jun 21;14(6):e1007124. doi: 10.1371/journal.ppat.1007124 (PMC6013022; doi:10.1371/journal.ppat.1007124)
Supplement: S2 Table — (DOCX) [file ppat.1007124.s012.docx]

**S2 Table. Oligonucleotide list**

|  | **name** | **sequence (5' --> 3')** |
| --- | --- | --- |
| Primer | HBV fwd | GAATTGATGACTCTAGCTACCTG |
|  | HBV rev | GAAACCACAATAGTTGCCTGATC |
|  | cccDNA fwd | CGTCTGTGCCTTCTCATCTGC |
|  | cccDNA rev | GCACAGCTTGGAGGCTTGAA |
|  | FEN1 ORF fwd | CTGTGGACCTCATCCAGAAGCA |
|  | FEN1 ORF rev | CCAGCACCTCAGGTTCCAAGA |
|  | FEN1 3'UTR fwd | GTTCAGCCTTGACCCACCTT |
|  | FEN1 3'UTR rev | TCTCTCAGCAGTTCCTCCCA |
|  | HPRT fwd | GCCCTGGCGTCGTGATTAGT |
|  | HPRT rev | CGAGCAAGACGTTCAGTCCTGTC |
|  | Pre-C fwd | TAGGCATAAATTGGTCTG |
|  | Pre-C Rev | GATTGAGATCTTCTGCGACGC |
|  | β-actin fwd | GACCTGACTGACTACCTCATGAAGA |
|  | β-actin rev | GGGGCCGGACTCGTCATACTCCTGC |
|  | FEN1 sequencing fwd | GTGTATGTCTTTGATGGCAAGC |
|  | FEN1 sequencing rev | TCACTGGGTGCATCAAGATAAG |
|  | hFEN-ΔC-1643 | GTGACCGGCTCACTCTCTTCAGCTAAGCGCAAGGAGCCAGAActcgagT |
|  | hFEN-ΔC-1644 | CTAGActcgagTTCTGGCTCCTTGCGCTTAGCTGAAGAGAGTGAGCCG |
|  | cccDNA sequencing | TTCTCGATACAGAGCTGAGGC |
| Probe | cccDNA Taqman | CTGTAGGCATAAATTGGT |
| RCA primer | RCA1 | AATCCTCACAATA*C*C |
| (* = modified with | RCA2 | GATGGGATGGGAA*T*A |
| phosphorothioate) | RCA3 | CCTATGGGAGTGG*G*C |
|  | RCA4 | GCAACGGGGTAAA*G*G |
|  | RCA5 | ATGCAACTTTTTC*A*C |
|  | RCA6 | TCCAAATTCTTTA*T*A |
|  | RCA7 | TAGAAGAAGAACT*C*C |
|  | RCA8 | AGAATATGGTGAC*C*C |
| FEN assay | template | GCGCACCAGCACCATGGTACCTCTGCCTAATC |
|  | quencher | GATTAGGCAGAGGTGTTGAAAAAGACATC |
|  | flap | GATGTGAAAAAGTTGCATGGTGCTGGTGCGC |
| FEN1 sgRNA | top strand | caccgAGCTGGCCAAACGCAGTGAG |
|  | bottom strand | aaacCTCACTGCGTTTGGCCAGCTc |
